# Mechanisms of foreign language learning anxiety and enhancement strategies among Chinese tertiary students: a grounded theory approach

**DOI:** 10.3389/fpsyg.2024.1512105

**Published:** 2025-01-17

**Authors:** Junxia Gao, Yanpeng Zuo

**Affiliations:** ^1^Department of Public Education, Guangdong Polytechnic of Environmental Protection Engineering, Guangdong, China; ^2^College of Education Science, South China Normal University, Guangdong, China

**Keywords:** foreign language learning anxiety, grounded theory, Chinese tertiary students, model of mechanisms, teaching strategies

## Abstract

Given the dynamic and contextual nature of foreign language learning anxiety (FLLA) and its impact on language acquisition, this study aims to gain a deeper understanding of the factors and mechanisms that underlie FLLA. Utilizing Nvivo 12 and grounded theory, the study conducts a coding analysis of interview data from tertiary students from 16 higher institutions in China, and reflection journals from two classes at a vocational college in China. The analysis identifies two core categories of anxiety: external sociocultural factors and internal self-regulation issues. The study constructs a model which indicates that external factors, such as maladaptation to the academic transition, disconnection between high school and university curricula, and intense academic competition, directly contribute to this anxiety. Intense academic competition raises students’ self-expectations, exacerbates time management difficulties, and further intensifies their anxiety. Additionally, a vicious cycle develops between students’ foreign language learning anxiety and their internal motivation and self-efficacy. To alleviate this anxiety, the study proposes teaching strategies to foster positive emotions, including enhancing self-regulation, instilling a growth mindset, promoting flow experiences, cultivating a positive self-concept, and creating a supportive classroom atmosphere. Future research should adopt a dynamic complexity theory perspective to explore trends in anxiety and its relationships with other affective factors, with the goal of developing more effective interventions.

## 1 Introduction

Foreign Language Learning Anxiety (FLLA), a form of situational anxiety, diverges from general anxiety due to its language-specific characteristic. FLLA is defined as the discomfort, tension, nervousness or worry that learners may experience during the process of acquiring and using a second or foreign language in both in-class and out-of-class settings (Gregersen and MacIntyre, 2014). Recent years have witnessed a surge in research on FLLA and its significant impacts on language acquisition (Jin et al., 2020; Toyama and Yamazaki, 2021; Hayasaki and Ryan, 2022).

FLLA is characterized by its dynamic and context-specific nature. Anxiety levels fluctuating across different periods and situations demonstrate the dynamism of FLLA (Sampson, 2019; Dewaele and Dewaele, 2020). This variability is evident across different timescales, for example, university students’ FLLA may exhibit ups and downs within a semester (Shirvan and Taherian, 2021) and can vary between classes and even within a single session (Kruk, 2018). Gkonou et al. (2020) used the metaphor of “rollercoaster” to describe such variation with contextual changes. Furthermore, FLLA is contextualized, with its context-specific nature resulting from interactions between individuals and their environments (Ushioda, 2009). FLLA levels differ across individuals based on factors such as cultural background and language proficiency (Horwitz, 2016). Interactions among teachers and students, peer dynamics, and the use of various instructional resources can either worsen or mitigate FLLA (Khatereh and Elahi, 2020).

To capture the dynamic and contextual nature of FLLA, various research methods have been employed. Quantitative scales, such as the Foreign Language Classroom Anxiety Scale (FLCAS) developed by Horwitz et al. (1986) or its shorter versions like the Short-form Foreign Language Classroom Anxiety Scale (S-FLCAS) by Botes et al. (2022) and the 17-item scale by Yuan (2023), are used to assess anxiety levels. However, given the multifaceted nature of FLLA, some researchers argue that scales alone may not capture the complexity of learners’ anxiety. Consequently, qualitative methods, such as interviews (Balkaya et al., 2020), content analysis (Piniel and Albert, 2018), idiodynamic approaches (Khatereh and Elahi, 2020), and physiological measures such as cortisol levels in hair samples (Dewey et al., 2018), have been utilized to gain deeper insights into FLLA.

Despite the progress made in FLLA, there remain areas for improvement. Existing empirical research has explored specific aspects of FLLA, such as academic background (Gopang et al., 2017), classroom discussion topics (Harputlu et al., 2018) and peer pressure (Laachir et al., 2022), but these studies often focus on overt dimensions of anxiety without delving into its underlying mechanisms. Furthermore, current studies in this field often involve homogeneous samples from specific regions, universities, or even particular classes or programs (Tao and He, 2021; Chen et al., 2022; Rohliah et al., 2023). The homogeneity limits our understanding of FLLA across diverse learner populations. Additionally, most studies target undergraduate students (Zhang and Han, 2024), with limited research on middle and primary school students (Dewaele and Li, 2022; Li and Li, 2023) and even less focus on vocational students. Given the equal importance of vocational education in the higher education system, as highlighted by the *Vocational Education Law of the People’s Republic of China* (The State Council, 2022), future research should expands to include vocational students for a more comprehensive understanding of FLLA across different educational contexts.

The recent and notable shift toward positive psychology in foreign language teaching highlights the influence of positive emotions upon language learning (Xu, 2020). Approaches grounded in positive psychology have been shown to effectively alleviate anxiety in language learning contexts (Jin et al., 2021).

To sum up, this study aims to address the gaps in the existing literature by exploring the underlying mechanisms behind FLLA experienced by Chinese tertiary students during their English learning. The study intends to analyze learning reflections and interview data from students across a variety of educational institutions in China by adopting grounded theory as a methodological approach, considering that grounded theory is a bottom-up approach that inductively generates concepts and propositions from raw data to explore core concepts of social phenomena and forms theories through the relationships among these concepts (Chen, 2000). The objective is to develop a deeper understanding of the factors that contribute to FLLA and to propose targeted strategies to alleviate this anxiety.

Furthermore, by involving vocational students in this study, a relatively under-researched group in the context of FLLA, this study aims to broaden the scope of FLLA research, offering new insights into how different learner populations experience and cope with language learning anxiety. The findings will provide valuable implications through the lens of positive psychology for teachers in creating supportive learning environments that foster both academic success and emotional well-being among students.

Specifically, this study will address the following two research questions:

1. What are the mechanisms behind the formation of FLLA among Chinese university students?
2. How can teaching strategies be designed from a positive psychology perspective to effectively alleviate FLLA among Chinese tertiary students?

## 2 Method

### 2.1 Research design

This study employs Procedural Grounded Theory developed by Strauss and Corbin (1998) to investigate the mechanisms underlying FLLA among Chinese tertiary students. This theory represents a refined application of traditional grounded theory, emphasizing a structured approach to data collection and analysis for the iterative development and validation of theoretical models. It focuses on elucidating the relationships and mechanisms among concepts while ensuring the logical coherence of the emerging theory (Strauss and Corbin, 1998).

The application of Procedural Grounded Theory in this research aims to construct a comprehensive theoretical model of FLLA. By analyzing the specific contexts and contents, the study seeks to uncover the underlying mechanisms contributing to this phenomenon, providing a thorough understanding of its complexities.

To achieve these objectives, the study utilizes NVivo 12 for qualitative data analysis, employing a three-tiered coding process: open coding, axial coding and selective coding. The research process involves analysis, organization, validation and saturation testing to ensure that the final model accurately reflects the complexity of FLLA among Chinese tertiary students.

### 2.2 Participants

Participants from two classes wrote reflective journals about their English learning experiences. They come from the first author’s home institution, a public higher education institution located in South China, where Yvonne, a senior English teacher with 12 years of teaching experience, has always encouraged her students to write reflections on their learning as part of a learning portfolio. This practice aimed to gain insights into the students’ learning experiences and affective states. Weekly reflective journals were not mandatory; however, students were encouraged to document instances of FLLA in their journals to give reflection on their genuine experiences in both classroom and self-directed learning contexts.

To comprehensively understand student reflections, the study selected classes with different backgrounds and types. Two classes were chosen for journal collection: Class One included 46 students majoring in Environmental Engineering Technology (High-to-Bachelor Bridging Program) with 199 reflections collected, In this program, students are high school graduates who should complete 3 years of vocational college courses and, after passing all required exams, transfer to a designated institution for 2 years of full-time undergraduate education to earn a bachelor’s degree. Class Two consisted of 41 students majoring in Occupational Health and Safety Technology with 129 reflections collected. In this class, some students come from high schools, and some from vocational high schools. All students study for three years to earn a graduation certificate.

For the interview phase, participants were selected through convenience sampling. The first author contacted teachers from various regions of China, whom she had met during her visiting-scholar period, and invited them to recommend students for interviews. Participants were chosen based on their responsible attitude, commitment and willingness to participate, rather than their language proficiency. To ensure diversity in the sample, 18 interviewees were selected from 16 different higher education institutions (including both undergraduate and vocational colleges) across various regions, disciplines, academic levels and genders. Senior students were excluded from the interviews due to their commitments to thesis, internship and job searching. The sample size was determined based on the principle of theoretical saturation. After analyzing data from 14 interviews, no new information emerged, and subsequent interviews with 4 additional participants yielded repetitive contents. Thus, saturation had been reached. Table 1 provides demographic details of the 18 participants.

**Table 1:** Participant demographic information.

No.	ID	Gender	Year	Institutions (2024 Shanghai Ranking^1^)	Major	City/Region
A1	CG	Male	Freshman	Guangdong University of Technology (100)	Electronics Information	Guangzhou/South China
A2	HG	Female	Freshman	South China Normal University (79)	Finance (Joint Program)	Foshan/South China
A3	ZG	Male	Freshman	South China Normal University (79)	Software	Foshan/South China
A4	GK	Male	Freshman	China University of Mining and Technology (77)	Geology	Beijing/North China
A5	ZW	Female	Freshman	Henan Institute of Science and Technology (339)	Fashion Design	Xinxiang/Central China
A6	LQ	Male	Freshman	Shanxi Agricultural University (216)	English	Jinzhong/North China
A7	ZB	Female	Freshman	Weinan Normal University (396)	English	Weinan/Northwest China
B1	CH	Male	Sophomore	Henan Institute of Science and Technology (339)	International Trade	Xinxiang/Central China
B2	LC	Female	Sophomore	Changchun Normal University (313)	Economics and Finance	Changchun/Northeast China
B3	WS	Male	Sophomore	Beijing Normal University (19)	English	Beijing/North China
B4	ZC	Female	Sophomore	Wenshan University (586)	Preschool Education	Wenshan/Southwest China
B5	ZM	Female	Sophomore	Hangzhou Dianzi University (97)	Intelligent Finance	Hangzhou/East China
C1	GG	Female	Junior	Guangdong Ocean University (300)	Accounting	Zhanjiang/South China
C2	ZC	Male	Junior	South China University of Technology (30)	Applied Chemistry	Guangzhou/South China
C3	DAI	Female	Junior	Wuhan University of Technology (52)	Journalism	Wuhan/Central China
D1	ZH	Female	Freshman	Guangdong Polytechnic of Environmental Protection Engineering	Environmental Engineering Technology	Foshan/South China
D2	YG	Male	Freshman	Guangdong Industry Polytechnic University	International Trade	Guangzhou/South China
D3	GU	Male	Sophomore	Yangquan Teachers College	Primary School English Education	Yangquan/Northwest China

^1^2024 Best Chinese Universities Ranking: https://www.shanghairanking.cn/rankings/bcur/2024 (with vocational colleges excluded from the ranking). A for freshmen; B for sophomores; C for juniors; D for vocational college students.

### 2.3 Data collection

This study employs both longitudinal and cross-sectional methods to comprehensively explore FLLA among students. First, longitudinal data collection took place over a 16-week semester across two classes taught by Yvonne, with students submitting weekly reflective journals. These reflections, written in either English or Chinese, documented students’ in-the-moment emotions, personal experiences, questions and insights related to English learning. By submitting their reflections voluntarily via an online learning platform, students helped mitigate recall bias (Li et al., 2024), and the platform facilitated systematic data collection and analysis.

Second, cross-sectional interviews were carried out. Prior to the interviews, participants were informed of the study’s purpose and encouraged to provide authentic and detailed responses. Interviews, conducted via WeChat voice, QQ voice or telephone, lasted between 40 to 90 min. During these interviews, participants were guided to recount specific anxiety-provoking situations during their English learning at both in-class and out-of-class contexts, with follow-up questions to gather more detailed information. The interviews utilized open-ended and semi-structured questions, such as: “Describe your emotional responses during university English classes and their causes,” “Have you experienced tension or anxiety during English learning?,” “What do you think are the causes of these emotions?” and “What methods do you believe teachers could use to help alleviate this tension or anxiety?”

The recorded interviews were transcribed and then reviewed by the participants, with revisions made based on their feedback to improve the reliability and validity of the data. 14 interview transcripts and the reflection journals from 46 students in Class One were used for the coding process. Additionally, the remaining 4 interview transcripts and reflective journals from Class Two were used for theoretical saturation testing.

## 3 Coding process

Data analysis followed Pocedural Grounded Theory approach proposed by Strauss and Corbin (1998), involving the stages of open coding, axial coding, and selective coding. This method facilitated a line-by-line examination of the data, the extraction of concepts, and the development of categories, ultimately leading to the construction of a theoretical model of FLLA mechanisms.

Two authors independently used NVivo 12 to code the data, and their open coding results were compared, yielding a Kappa coefficient of 97.3%, which indicated high inter-coder reliability. Any discrepancies were discussed and resolved to ensure accuracy. Throughout the coding process, the researchers maintained memos to document thoughts and insights related to categories and their mechanisms, as well as to explore theoretical directions and practical implications of the study’s findings.

### 3.1 Open coding

During the open coding phase, researchers read the original texts sentence by sentence and broke them down into discrete parts. The goal was to identify initial concepts through close examination. Concepts with similar meanings are grouped and consolidated into preliminary categories (Strauss and Corbin, 1998).

In this study, considering the English learning pressures faced by students in the “High-to-Bachelor” Bridging Program, we analyzed the reflection journals from Class One (totaling 32,541 words). Together with the learning reflections, interview data from 14 respondents (totaling 20,874 words) were imported into NVivo 12 for detailed line-by-line reading and coding.

To ensure the systematic nature of open coding, interview data were numbered accordingly. For instance, C1-1 refers to the first sentence of Respondent C1’s discourse, while E1 denotes the first sentence selected from the students’ reflection journals. After excluding irrelevant material, a total of 178 relevant original statements were identified. For example, the statement “The major courses are extensive and difficult. They encroach on my English learning time and induce insufficient time for English study” was coded as “conflict of learning time among courses.” This process resulted in 58 initial concepts.

Through iterative comparison, we further refined, abstracted and synthesized the initial concepts describing similar ideas. For instance, “Anxiety over vocabulary pronunciation,” “Insufficient vocabulary accumulation,” and “Unstable vocabulary spelling” were all related to vocabulary acquisition and were therefore consolidated into the category “Vocabulary acquisition barriers.” Ultimately, 58 initial concepts were distilled into 24 preliminary categories, as detailed in Table 2.

**Table 2:** Concepts and categories derived from open coding.

Original statements	Initial concepts	Preliminary categories
E150 I have a tight schedule every morning, and at night, I feel anxious about the unfinished tasks.	Over-scheduling	F1 Overload in planning
A6-2 Review time before exams is short, so everyone feels anxious.	Insufficient review time	
A2-3 The major courses are extensive and difficult. They encroach on English learning time and induce insufficient time for English study.	Conflict of learning time among courses	F2 Learning time conflicts
E144 We are busy preparing for the vocational skill competition, so I am afraid I might fall behind in English classes when I return.	Conflict between competitions and learning English	
A6-2 English classes at university are full of professional terms and the full English instruction makes it hard to follow.	Changes in the language of instruction	F3 Changes in teaching modes
B3-7 Unlike the repetitive exercises in high school, there are many group activities in College English courses.	Changes in teaching activities	
B3-1 I am not familiar with the content taught by professors, which makes me quite nervous.	Changes in course content	F4 Course content adjustments
B2-8 There are many topics covered in one class.	Increased volume of knowledge	
A4-1 There is no new content; it is the same as what we studied in high school.	Similarity between high school and college-level English	F5 Mismatch between English courses and personal needs
B2-5 Oral English skills are necessary for future work, but we have not had opportunities to practice them in class.	Disconnection between teaching modes and future work needs	
E27 Learning English is very difficult for me because I studied Japanese in high school.	Disconnection between past and present language courses	F6 Disconnection between high school and university education
C2-1 When giving an English presentation, students who did not have oral exams in high school are more nervous than those who did.	Disconnection in speaking skills development	
B5-1 I feel anxious because I put in the same effort in English as in other subjects, but I have not seen a corresponding improvement in my grades of English.	No improvement in grades	F7 Worries about grades
E50 My English grades have always been poor.	Poor English grades	
E92 I took the PRETCO-A^1^ and I am not sure about the result, which causes anxiety.	Uncertainty about exam results	F8 Concerns about proficiency tests
E60 I feel uncertain about how to prepare for the CET-4^2^.	Worries before the CET-4 exam	
A6-5 I am more concerned about not failing the final exams.	Concern about passing exams	F9 Anxiety about course exams
A2-1 I mainly feel anxious before exams.	Pre-exam worries	
C2-4 Poor exam performance means not performing as well as others, since admissions are based on rankings.	Ranking system	F10 Concerns about rankings
A6-7 In high school, we were told that exams and rankings are extremely significant in university.	Emphasis on rankings	
B5-3 My classmates with similar performance in other subjects have better grades in English, so their rankings are much higher than mine, which makes me feel anxious.	Comparison with peers	F11 Peer comparison
E95 Seeing others win awards in English speech and writing competitions makes me regret not becoming proficient in English.	Regret over English proficiency	
B4-2 I feel nervous and uneasy during English classes.	Nervousness	F12 Fear of the subject
D2-3 Fear contributes significantly to my anxiety.	Fear	
B4-4 I try to avoid English classes and English learning.	Avoidance of English learning	F13 Academic burnout
B167 I have a strong aversion to learning English.	Resisting English learning	
A2-4 I make many errors in pronouncing individual letters and struggle with phonetic symbols.	Anxiety over vocabulary pronunciation	F14 Vocabulary acquisition barriers
B1-7 I am anxious because there are many new words and terms in Business English.	Anxiety over vocabulary acquisition	
E7 Communicating in English makes me feel challenged.	Difficulty with oral communication	F15 Oral communication anxiety
B1-2 Poor pronunciation makes it hard for others to understand me.	Poor pronunciation	
B3-8 The variety of accents in listening materials creates pressure.	Difficulty in understanding varied accents in listening	F16 Difficulties in listening comprehension
C1-6 Listening is relatively weak; I make many mistakes and have trouble understanding.	Anxiety over listening proficiency	
A2-5 I feel I cannot reach the level required for IELTS or CET-4. It is difficult for me to improve my writing skills.	Inability to meet higher standards	F17 Difficulties in meeting higher standards
A3-3 It is hard to write creative and excellent sentences in essays.	Stagnation of writing skills	
E18 I am anxious because I cannot use complex sentence correctly.	Difficulty with grammar usage	F18 Struggles with grammar mastery
E7 I cannot distinguish between restrictive and non-restrictive relative clauses.	Poor command of relative clauses	
A4-1 Long and complex sentences, which were rare in high school, are now common in College English.	Fear of long and complex sentences	F19 Reading difficulties
E135 The reading comprehension exercise last week was not difficult, but because of the new words, I could not find the correct answers.	Low accuracy in reading comprehension	
E75 My poor English prevents me from participating in class activities.	Lack of participation in activities	F20 Unsatisfactory class performance
B1-2 I am hesitant about sitting in the front rows.	Avoiding front row seating	
C1-1 I get nervous when I do not know what the teacher is discussing and cannot answer questions.	Anxiety about being questioned	F21 Fear of being questioned
D2-2 I am very afraid of being asked questions in class, as I often cannot answer and feel embarrassed.	Fear of not answering questions	
D1-2 Speaking English in front of others makes me very nervous.	Nervousness about public speaking	F22 Public speaking anxiety
C1-3 Presenting assignments in English makes me nervous.	Nervousness about presentations	
D2-2 I feel some pressure from English assignments.	Pressure from English assignments	F23 Assignment overload
D1-1 English assignments add some pressure.	Stress from English assignments	
D1-4 My language abilities are insufficient, so I am anxious about it.	Poor English skills	F24 Negative self-evaluation
E74 My English foundation is weak; I quickly forget words and grammar points and have trouble remembering much.	Weak English foundation	

Due to space limitations, only a subset of the concept codes is presented in this table. ^1^Practical English Test for Colleges, Rank A (PRETCO-A) is a national English proficiency test for vocational college students. ^2^College English Test, Band 4 (CET-4) is a national English proficiency test for university students.

### 3.2 Axial coding

Axial coding, the second stage of the coding process, aims to analyze and compare the initial categories identified from open coding to establish their connections, and refine them into more cohesive primary categories (Strauss and Corbin, 1998). During this stage, 24 preliminary categories were further integrated. For example, freshmen exhibit noticeable maladaptation due to “Changes in teaching modes” and “Course content adjustments,” so these preliminary categories were consolidated into the primary category of “Maladaptation to the transition period.” Ultimately, the factors influencing FLLA among Chinese tertiary students are distilled into 7 primary categories, as presented in Table 3.

**Table 3:** Primary categories derived from axial coding.

Primary categories	Preliminary categories	Description
Z1 Difficulties in Time Management	F1 Overload in planning	Heavy course load and excessive extracurricular activities lead to less time for English study.
	F2 Learning time conflicts	Conflicts between time allocated to skill competitions and that to major courses and English study create pressure.
Z2 Maladaptation to the Transition Period	F3 Changes in teaching modes	Changes in teaching modes, including classroom activities, teaching pace, objectives, and teaching language, cause anxiety during the transition period.
	F4 Course content adjustments	The increased volume and emphasis on systematic knowledge in college English courses contribute to the transitional anxiety.
Z3 Incoherence in the English Curriculum Structure	F5 Mismatch between English courses and personal needs	Discrepancies between English course content and students’ personal and career needs lead to anxiety or concern.
	F6 Disconnection between high school and university education	The disconnection of the importance of listening and speaking skills and the dissonance with previous Japanese language courses lead to anxiety.
Z4 Intense Academic Competition	F7 Worries about grades	Unsatisfactory grades in various English courses create a psychological burden.
	F8 Concerns about proficiency tests	The need to pass English proficiency tests in university causes pressure.
	F9 Anxiety about course exams	Pre-exam pressure and post-exam disappointment make students anxious.
	F10 Concerns about rankings	The connection between exam scores and rankings, GPA, admissions and awards contributes to students’ pressure.
	F11 Peer comparison	Comparing English proficiency, grades, and task completion quality to peers causes stress.
Z5 Weakened Will to Learn	F12 Fear of the subject	Negative emotions, such as nervousness, distress, and fear, arise from English classes and learning.
	F13 Academic burnout	Negative emotions lead to aversion and avoidance of English classes.
Z6 Widening Gap Between Cognitive Demands and Abilities	F14 Vocabulary acquisition barriers	Insufficient vocabulary, pronunciation errors, and spelling difficulties cause significant concerns.
	F15 Oral communication anxiety	English communication, including pronunciation, public speaking, and verbal exchanges, leads to anxiety.
	F16 Difficulties in listening comprehension	Challenges with vocabulary, rapid speech, diverse accents, and lack of practice result in listening anxiety.
	F17 Difficulties in meeting higher standards	Difficulties in meeting higher writing standards, including vocabulary use, logical reasoning and cultural differences, lead to frustration.
	F18 Struggles with grammar mastery	Struggles with understanding and applying grammar concepts and rules contribute to anxiety.
阅读	F19 Reading difficulties	Problems with vocabulary, syntax, or specialized terms hinder comprehension and cause difficulty.
Z7 Low Self-Efficacy	F20 Unsatisfactory class performance	Lack of confidence or courage to participate in class activities results in dissatisfaction with performance.
	F21 Fear of being questioned	Fear of being questioned arises from a poor self-perception and a lack of confidence.
	F22 Public speaking anxiety	Public speaking in English classes causes nervousness.
	F23 Assignment overload	Cognitive overload comes from difficult assignments or heavy class tasks.
	F24 Negative self-evaluation	Negative self-assessment due to poor English performance affects students’ self-efficacy.

### 3.3 Selective coding

Selective coding is the final phase of the coding process. During this phase, a core category is identified that unifies and integrates the primary categories developed during axial coding. The logical relationships between core categories and primary categories are closely examined to build a cohesive theoretical narrative (Chen, 2000).

Recognizing that FLLA is a multifaceted psychological construct (Young, 1991), shaped by both internal learner factors (Jiang and Dewaele, 2019) and sociocultural environmental influences (Douglas Fir Group, 2016), this study integrates seven primary categories into two core categories:

The three primary categories-maladaptation to the transition period, incoherence in the English curriculum structure, and intense academic competition-encompass external factors such as sociocultural background, educational policies and societal values. These factors collectively shape the challenges faced by tertiary students in English learning. Therefore, these primary categories are classified under the core category of “sociocultural factors.”

The four core categories-weakened will to learn, low self-efficacy, the widening gap between cognitive demands and abilities, and difficulties in time management-relate to aspects of learners’ self-regulated learning. Specifically, learning will affects students’ persistence; cognitive gaps involve the alignment between learning strategies and cognitive demands; self-efficacy impacts students’ confidence in handling learning tasks; and time management is crucial for effective planning and execution of learning activities. Since all four aspects are related to students’ self-regulated learning abilities, they are categorized under the core category of “individual self-regulated learning troubles.”

**Figure 1:**
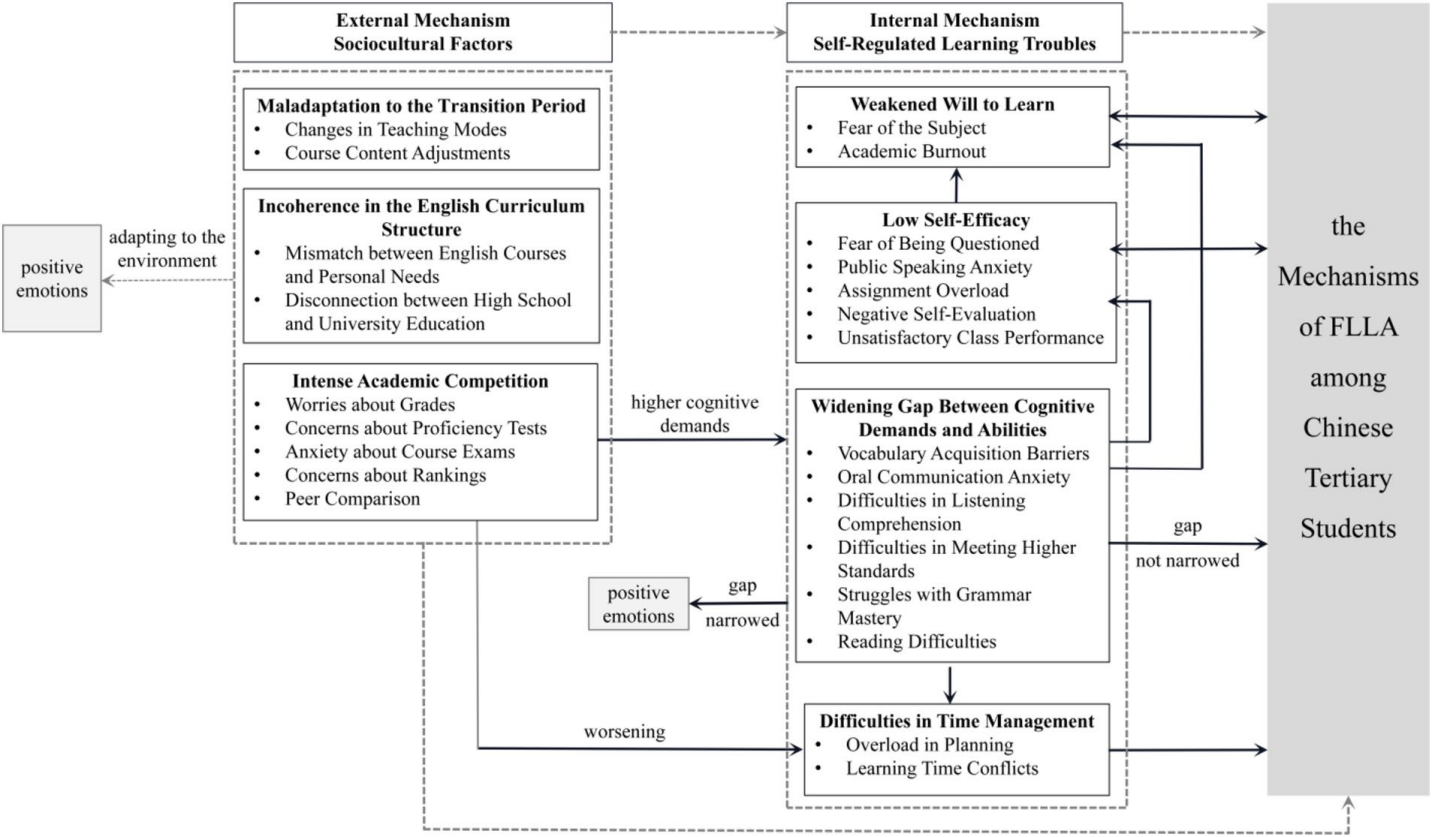
Framework of the mechanisms underlying the formation of FLLA among Chinese tertiary students (Dashed lines represent the relationships between core categories and the research topic, while solid lines indicate the relationships among primary categories, as well as between primary categories and the research topic).

Thus, the two core categories-sociocultural factors as an external mechanism and individual self-regulated learning troubles as internal mechanism-provide a comprehensive framework for understanding the mechanisms underlying the formation of FLLA among Chinese tertiary students. The relationships between the core and primary categories are illustrated in Figure 1.

### 3.4 Theoretical saturation test

Theoretical saturation is achieved when additional data no longer contribute new concepts or insights, indicating that the theoretical model has reached a state of saturation (Strauss and Corbin, 1998).

In this study, we used 14 interview transcripts and the learning journals from 46 students in Class One for the coding process. To assess theoretical saturation, we also analyzed the additional 4 interview transcripts and reflective journals from Class Two. The analysis revealed no new concepts or categories, and no novel patterns emerged in the relationships among existing categories. This indicates that the theoretical model has successfully passes the theoretical saturation test.

## 4 Results

The coding results suggest that both sociocultural factors and students’ challenges in self-regulated learning contribute to FLLA among Chinese tertiary students. FLLA emerges from the interplay between sociocultural factors and self-regulation difficulties. By systematically analyzing the relationships among original statements, preliminary categories, core categories, and the research theme, a detailed relational structure was developed: on one hand, sociocultural factors directly trigger FLLA; on the other hand, self-regulated learning troubles, resulting from sociocultural factors, induce FLLA among Chinese tertiary students.

### 4.1 Sociocultural environment as an external mechanism directly triggers FLLA

Traditional psychology and positive psychology primarily view learners’ emotions as individual attributes, with sociocultural background serving only as an influencing factor. In contrast, research from a sociocultural perspective asserts that emotions have both individual and sociocultural dimensions and should be explored within their sociocultural context (Qin et al., 2022). The findings of this study indicate that the sociocultural environment—including maladaptation to the transition period, incoherence in the English curriculum structure and intense academic competition—directly contributes to FLLA among Chinese tertiary students.

#### 4.1.1 FLLA arises from maladaptation to the transition period

The study reveals that freshmen face significant challenges in English learning during the transition from high school to university, primarily due to differences in teaching methods and course contents. The academic transition period, often referred to as the “anxiety period” for freshmen (Brady and Allingham, 2005), encompasses the initial phase of moving from secondary school to higher education. This includes the “freshman adjustment period” during the first semester and the “second transition period” from the second to the third semester (Petriwskyj, 2010). During this time, students must adjust from the repetitive, exam-oriented education model of high school to the more open and dynamic teaching approaches at university. This transition often involves difficulties in adapting to the cognitive and behavioral patterns required in higher education institutions (Song, 2007).

Upon entering university, students frequently experience confusion and anxiety due to the significant differences in English teaching modes between high school and university. English teaching modes refer to systematic instructional frameworks that encompass learning objectives, instructional activities, and teacher-student interactions (Hao and Xu, 2003).

In terms of learning objectives, the *National English Curriculum Standards for General High Schools* stipulates that English classes in high school aim to develop students’ language abilities, cultural awareness, critical thinking and learning capabilities (Ministry of Education, 2020). However, the intense focus on the Gaokao makes those classes prioritize exam-oriented education, focusing on students’ test scores and exam performance. This “repeated drilling” approach contrasts sharply with the focus of English teaching in colleges, which “*goes beyond textbook knowledge and emphasizes the expansion of knowledge and the holistic development of students’ competencies*” (*Respondent A6*). This shift in teaching focus necessitates a period of adjustment for freshmen. Moreover, in high school, teaching often emphasizes repeated explanations and practice of knowledge points, and relies heavily on the mother tongue for better comprehension. In contrast, “*classes are faster-paced and taught exclusively in English*” (*Respondent B3*). The rapid pace and all-English instruction in English classes in universities require students to abandon their previous reliance on repetitive practice and native language, which can lead to adaptation difficulties. Furthermore, the teacher-student interaction dynamics also change significantly. In secondary education, teachers usually dominate the teaching process, controlling the content and timing, which fosters a dependency mindset among students. However, “*English classes in colleges involve more group work, with higher demands for student autonomy and collaborative learning. This weakens the teacher’s dominant role in the classroom*” (*Respondent B3*). This transition from a highly controlled environment to one that emphasizes self-management and collaborative learning can be challenging for students.

Another challenge is the continuity of course contents. In high school, course contents are often designed around examination objectives to ensure students achieve high scores on the Gaokao, which results in a focus on certainty and standardization with a singular correct answer. In contrast, university courses emphasize a more systemic and logical approach to knowledge, covering broader and deeper contents. For example, “*Business English involves substantial professional knowledge in finance and trade, rather than just language learning*” (*Respondent C3*). This shift in course contents, while offering new learning opportunities, demands that students overcome greater cognitive and skill-based challenges.

In summary, new students face challenges in adapting to the English teaching modes at higher education institutions, including the transition from exam-focused education to holistic skill development, shifting from a reliance on native language to full English instruction, moving from teacher-directed to autonomous and collaborative learning, and adjusting to the increased complexity and challenge of course contents. These factors collectively contribute to the confusion and anxiety experienced by freshmen.

#### 4.1.2 FLLA arises from incoherence in English curriculum structure

Interview data reveal that the incoherence in the English curriculum structure between secondary and higher education manifests in two primary ways: one is the discrepancy between learning objectives and students’ personal development needs, and the other is the discontinuity in language course offerings.

English courses in colleges fail to meet the needs of students’ career development and incorporate practical, real-world English skills, which leads to anxiety and frustration among students. Education is expected to serve learners’ individual lives, and the curriculum should meet students’ personal needs, interests, and academic goals (Zhang et al., 2013). However, in practice, English courses in colleges often fail to align with students’ needs of career development. “*Oral communication is crucial for our future jobs, but our English classes are so traditional that we have not had the opportunity to practice speaking English*” (*Respondent B2*). Some teachers continue to use traditional, teacher-centered methods, unable to incorporate English knowledge relevant to real-world work scenarios. Consequently, learners may question the practicality of the course, which generates anxiety. “*The textbook and the teacher’s lectures have not provided us with any business English knowledge. But as vocational students, we need knowledge about business and the workplace. My future career development requires more options, and there’s a section in the national vocational college skills competition involving business English too*” (*Respondent D3*).

The preference for other foreign languages, such as Japanese, over English in the Gaokao due to the lower difficulty results in challenges and anxiety for students who must study English again in colleges. The *Regulations on Admissions Work for General Higher Education Institutions,* released by the Ministry of Education in 2024, specifies that candidates can choose from English, Russian, Japanese, French, German, and Spanish for the Gaokao (Ministry of Education, 2024). National policies also stipulate that the difficulty level of foreign language exams in less commonly taught languages should be 5 to 10 percentage lower than that of English (Ministry of Education, 2018). For instance, Japanese exams are less challenging with fewer vocabulary requirements. Therefore, Japanese offers greater potential for score improvement in a shorter period when compared to English. That is why it has become a preferred choice for many secondary school students. The number of students choosing Japanese for the Gaokao increased from 9,600 in 2016 to 240,000 in 2021, a twenty-five-fold rise (Zhang and Qin, 2020). In Class One, 9 out of 46 students (19.6%) were Japanese exam candidates. Due to their choice of language for the Gaokao, these students face difficulties and challenges when required to study English again in college. The substantial gap between Japanese learning in high schools and English courses in colleges causes anxiety for these students. “*After studying Japanese for three years, I thought I could avoid my weakest subject in college, but unexpectedly, English became a compulsory course*” (*Respondent E29*). “*After learning Japanese, I completely forgot English. I tried my best to improve my English in college, but with no foundation, I felt completely lost and anxious”* (*Respondent E43*).

The abolition or reduced importance of English listening in the Gaokao in some provinces causes students from these regions to struggle with listening comprehension, which causes anxiety about catching up with their peers. In provinces such as Henan, Shanxi, and Liaoning, English listening has been abolished or does not contribute to the overall score. Under the influence of the educational philosophy “college entrance exam as a guiding tool,” this shift diminishes the importance of listening skills, leaving students’ listening and speaking skills rather poor. However, College English emphasizes the comprehensive development of language skills, with listening becoming increasingly important. For the students from these provinces, the discontinuity in listening leads to heightened anxiety. “*I came from a province where listening was not tested, so there was no practice with listening in high school. Now it makes it difficult for me to catch up with others in listening comprehension. It’s very stressful for me*” (*Respondent A4*).

#### 4.1.3 FLLA arises from intense academic competition

Anxiety is both an individual psychological phenomenon and a social construct that develops from social relationships (Prior, 2019). The cultural and social contexts in which learners are situated profoundly influence their experiences of anxiety (Kim, 2022). In China, education is regarded as a major tool for social stratification, and intense academic competition is a common source of academic anxiety (Gu and She, 2020).

Exams, a key factor leading to FLLA, are one manifestation of the intensity of academic competition. Given the sharp contrast between China’s large population and relatively scarce educational resources, studying hard is perceived as an effective way to achieve social status and economic success. The Gaokao, as a selective examination system, amplifies the evaluative function of education with greater emphasis placed on scores, which in turn shapes learners’ attitudes toward exams and grades. Significant exams, such as final exams and proficiency tests, are crucial external factors contributing to FLLA (Hu et al., 2024). Students in this study reported feeling anxious due to uncertainty about exam preparation (*Journal E60*), unfamiliarity with exam formats (*Journal E160*), and worries about poor exam outcomes (*Journal E92*). Additionally, anxiety arises when peers pass proficiency tests while they do not (*Journal E150*). As one student noted, “*The pressure before exams is immense. From elementary to high school, we were constantly reminded of the critical importance of exams, so even now, as a university student, I am still unconsciously aware that exams are crucial*” (*Respondent A6*).

Furthermore, the widespread adoption of the Grade Point Average (GPA) system intensifies academic competition, which creates significant psychological pressure on students striving to improve their academic performance. GPA, which assesses student learning based on course grades and credit hours, is commonly used in colleges and universities nationwide. This system facilitates the comparison of academic performance among students from different disciplines and institutions, aiding in awards, further education, study abroad opportunities, and job applications. One student commented, “*I believe that GPA ranking is indeed the primary source of anxiety for students at our stage because scholarships and awards are linked to GPA rankings.”* In the context of graduate school admissions based on recommendations, both GPA and rankings play pivotal roles. The stress often arises from the intense competition to achieve better academic performance. “*We strive to improve our classroom participation to boost participation grades, and we dedicate significant effort to studying in order to enhance our exam scores*” (*Respondent C3*).

Additionally, academic competition is not solely driven by individual effort; it also involves comparisons with peers. Students frequently engage in comparisons with their classmates, whether it’s assessing English proficiency levels (*Respondent A2*) or evaluating academic rankings (*Respondent B1*). These comparisons can exacerbate learning anxiety. For instance, a student might express anxiety when observing that “*My classmates with similar performance in other subjects have significantly higher English grades and rankings*” (*Respondent B5*). Such comparisons can create a sense of pressure and unease.

### 4.2 Self-regulation learning troubles as internal mechanism induce FLLA

This section explores how external sociocultural factors interact with internal mechanisms, contributing to FLLA.

Sociocultural factors, as external triggers, pose significant challenges to self-regulated learning among Chinese tertiary students. For example, the widening gap between cognitive demands and individual capabilities, weakened will to learn English, low self-efficacy, and difficulties in time management, all of which exacerbate FLLA.

Self-regulated learning (SRL) refers to the process in which learners proactively use and manage metacognitive, motivational and behavioral strategies to ensure successful learning outcomes and achieve their goals. SRL is multifaceted, including dimensions such as time management, motivation, methods, performance, environment and social factors (Schunk and Greene, 2017).

#### 4.2.1 Time management difficulties intensified by academic competition triggers FLLA

From the perspective of study time, learners with strong self-regulation skills are typically adept at efficient time planning and management. However, Chinese university students often face significant challenges in time management due to intense academic competition. To vie for awards, scholarships and graduate school admissions, students must invest substantial time and effort in coursework (*Journal E16*), extracurricular activities (*Journal E3*) and exam preparation, which consequently reduces the time available for English language study. Additionally, the complexity of course content and conflicting schedules (*Respondent A2*) frequently encroach on time allocated for learning English (Yan, 2022).

Under the pressure of heavy academic and extracurricular demands, students often struggle to effectively allocate their study time, exacerbating their learning anxiety. Research indicates a negative correlation between time management skills and academic anxiety, with poor time management contributing to increased learning stress (Ghiasvand et al., 2017). As mentioned in the reflection journal, “*I felt quite disheartened since I did not accomplish the study plan I had set for myself this week. My time management skills appeared insufficient, so I failed to plan and utilize my time effectively*” (*Journal E116*).

#### 4.2.2 The gap between cognitive demands and abilities, widened by intense academic competition, triggers FLLA

A significant contributor to FLLA is the discrepancy between learners’ cognitive demands and their language proficiency. When faced with challenging language tasks, students with low language proficiency experience heightened anxiety (Goñi Osacar and Lafuente Millán, 2021). Our study reveals that regardless of the level of university or the English proficiency of Chinese students, they consistently encounter difficulties across all aspects of language skills, including listening, speaking, reading, writing, vocabulary, and grammar. This mismatch between the cognitive demands of language learning tasks and learners’ personal abilities can affect their perceived control over tasks and subsequently trigger various academic emotions (Pekrun, 2006).

Scholarships, GPA and various certifications from proficiency tests or skill competitions are considered standards for marking “outstanding” students in China’s higher educational institutions. Students feel pressured to continuously enhance their language skills to remain competitive for this standards. This high level of self-expectation and stringent standards further exacerbates language-related cognitive anxiety. For instance, *Interviewee C1* reported, *“CET-6 is meaningful for me, but its listening comprehension is so challenging for me that I could not catch what the speakers said and got poor scores for this part. I felt so anxious at that time*,” and *Interviewee A2* mentioned, *“The writing in the Gaokao is not a problem for me, but now I find it hard to reach the level required for IELTS essays”*.

Vocational college students face a different set of challenges. Insufficient language ability amplifies the perceived difficulty of learning tasks. The gap between their self-perceived language proficiency and the high-level expectations contributes to a sense of inadequacy, further intensifying their anxiety. For instance, students in Class One experience pressure from the English entrance exam, which determines their eligibility for further bachelor education in the designated college. The grammatical and vocabulary requirements of the exam contribute to their stress and anxiety. Some students express their concerns: “*I am very anxious. I’m not very familiar with the vocabulary, and my grammar usage is incorrect*” *(Journal E1)*. Others mentioned feeling lost in class and worried about upcoming exams: “*I feel a bit lost in class. I’m also worried about tomorrow’s PRETCO-A, fearing that it will be a failure*” *(Journal E25)*. Another student described: “*I do not have much vocabulary in my mind, so when I am asked to create sentences or write, I feel like a blank slate*” *(Journal E43)*.

#### 4.2.3 Low self-efficacy and weakened will to learning English arising from the gap trigger FLLA

Cognitive Load Theory (Sweller, 1988) asserts that cognitive overload occurs when the difficulty of learning tasks exceeds the cognitive resources available to learners. Intrinsic cognitive load (ICL) is influenced by the complexity of the learning content and materials, as well as learner’s background knowledge. During the process of foreign language learning, factors such as the difficulty of learning tasks (*Journal E25*), the complexity of class questions (*Respondent C1*), grammatical intricacies (*Respondent B5*), extensive vocabulary requirements (*Respondent A2*), challenges in applying advanced vocabulary and complex sentence structures in writing (*Respondent A3*), fast-speed (*Respondent C1*) and diverse accents (*Respondent B3*) of listening materials can lead to ICL when the difficulty of language skill tasks exceeds the learner’s prior knowledge.

This ICL often brings about learning anxiety. In this case, the extent to which learners can effectively narrow the gap between the language cognitive demands and their abilities hinges on the their capacity to deploy self-regulation skills. According to Priestley et al. (2015), learners’ ability to mobilize self-regulation—by interacting with their potential, available resources and environmental factors to take proactive actions—is crucial for achieving learning outcomes. Self-regulated learning is a volitional process that requires learners to exert considerable effort and perseverance to achieve their learning goals (Cheng, 2023). Thus, learning will is an essential psychological tool for overcoming obstacles and achieving learning objectives.

Students with strong learning will, who face the gap between cognitive demands and their language proficiency, tend to view anxiety as a “positive impetus” and transform it into “autonomous learning motivation” (*Respondent A3*). They utilize their self-regulation abilities to leverage various resources and adopt appropriate learning strategies according to different tasks, such as watching American TV shows to foster interest (*Respondent B5*), accumulating useful vocabulary and sentence structures from exemplary texts (*Respondent A2*), and proactively seeking supplementary materials after class (*Respondent B3*).

Conversely, there is a significant negative correlation between foreign language anxiety and self-efficacy (Zhou et al., 2023; Chaves-Yuste et al., 2024). When faced with a gap in language proficiency, students experiencing FLLA often display low self-efficacy. This low self-efficacy leads to negative self-evaluation and self-doubt, as learners may feel that their “*language abilities are insufficient*” (*Respondent D1*) and their “*language foundation is weak*” (*Journal E74*). Such perceptions undermines their confidence. Consequently, they exhibit poor classroom performance, such as “*fear of participating in classroom activities*” (*Journal E76*), “*avoidance of sitting at the front of the classroom*” (*Respondent B1*), and “*fear of answering questions*” (*Journal E191*).

Self-efficacy influences students’ ability to self-regulate their learning. Those with low self-efficacy lack confidence in their learning abilities, perceiving difficulties as reflections of inadequacy. This diminishes their motivation and weakens their willpower, making them more likely to abandon their learning goals (Meng and Li, 1999). For example, one student reported, “*I listen attentively in class, but I really cannot understand when the teacher or classmates read English sentences. It’s very distressing*” (*Journal E59*). This statement reflects how some students, with weak learning resolve, struggle to bridge the proficiency gap, experiencing fear (*Respondent B4*), anxiety (*Respondent* D2), or even distress (*Journal E77*) during English classes. This can result in avoidance (*Respondent B4*), resistance (*Journal* E167), or rejection (*Journal E183*) of English classes, which triggers learning burnout and increased anxiety.

### 4.3 Enhancement strategies

This study employs grounded theory to explore and construct a model illustrating the mechanisms of FLLA among Chinese tertiary students. The model underscores the dynamic, complex and multifaceted nature of FLLA, analyzed through sociocultural factors as external mechanisms and learners’ SRL troubles as internal mechanisms. This model highlights the interplay between the factors contributing to FLLA. External sociocultural factors, such as maladaptation to the transition period, incoherence in the English curriculum structure, and intense learning competition, directly trigger FLLA.

These external sociocultural factors, in turn, affect learners and ultimately lead to self-regulation difficulties in learning. Specifically, intense academic competition drives students to raise their self-expectations, which increases the challenges associated with time management and widens the gap between cognitive demands and actual abilities. When this gap becomes difficult to bridge, FLLA intensifies, the will to learn English diminishes, self-efficacy declines, and thus a vicious cycle ensues.

According to the Broaden-and-Build Theory, positive emotions such as joy, interest, enjoyment and contentment can “undo lingering negative emotions,” “fuel psychological resiliency” and “improve emotional well-being” (Fredrickson, 2001). Therefore, based on the above-mentioned findings, it is recommended to foster positive emotions via English teaching practice to alleviate FLLA among Chinese tertiary students. This can provide valuable insights for improving teaching strategies to enhancing the effectiveness of English language instruction.

#### 4.3.1 Integrating learning strategies into classroom teaching to improve self-regulation capabilities

Some interviewees indicated that appropriate learning methods and strategies can alleviate the discomfort and anxiety associated with the transition period from high school to university. Effective use of learning strategies, such as metacognitive strategies, emotional regulation strategies, and time management strategies, is crucial for enhancing students’ self-regulated learning abilities. These strategies benefit students’ academic resilience under pressure (Mohan and Verma, 2020), helping them better manage FLLA due to intense academic competition.

#### 4.3.2 Strengthening value-added assessment to instill a growth mindset

Unlike traditional assessment methods used in secondary education, value-added assessment focuses on students’ starting points and their progress throughout the learning process. Teachers should place emphasis on value-added assessment instead of exams and scores to guide students to concentrate on the learning process and personal growth. Instilling a growth mindset through this approach can effectively alleviate FLLA among tertiary students (Barber, 2023).

#### 4.3.3 Increasing task control to enhance learner flow

Some interviewees noted that excessive difficulty in language tasks can lead to tension and anxiety. The Control-Value Theory suggests that the cognitive quality of learning tasks (i.e., task difficulty) affects students’ sense of control over these tasks, which in turn influences their academic emotions (Pekrun, 2006). By incorporating group-based collaborative tasks, establishing online courses to support personalized self-directed learning and creating learning communities, teachers can enhance students’ sense of control and self-efficacy. These measures contribute to increasing students’ enjoyment in language learning activities, which helps reduce FLLA (Xu and Zhao, 2024).

#### 4.3.4 Encouraging students to keep learning journals to build a positive self-concept

Teachers can guide students in keeping learning journals to record their learning progress and positive experiences. By developing students’ self-affirmation and positive self-concept, teachers can support students in maintaining their learning satisfaction and motivation, which is critical for alleviating FLLA due to intense academic competition.

#### 4.3.5 Employing digital teaching tools to create an enjoyable English classroom

Some interviewees emphasized that a relaxing and supportive language learning environment can effectively reduce anxiety and unease. The classroom environment and teacher factors significantly impact foreign language enjoyment (Botes et al., 2022). Teachers can empower classroom activities by utilizing such AI tools as ChatGPT and ERNIE Bot, as well as other digital teaching platforms like Bodoudou and Quizlet. This helps create an enjoyable and harmonious classroom atmosphere and improve students’ foreign language learning experience with the aim of reducing FLLA.

## 5 Discussion

To explore the mechanisms behind FLLA among Chinese tertiary students, this study employs grounded theory to analyze reflection journals and interview data from students across different levels of institutions in China. The results indicate that FLLA is primarily driven by sociocultural factors that influence students’ academic experiences and self-regulation in language learning. These findings not only confirm previous conclusions but also introduce novel insights into the complex nature of FLLA.

One of the key findings of this study is that FLLA among Chinese students primarily originates from their sociocultural environment. This finding aligns with Peng and Liu (2024) who emphasized the relationship between foreign language anxiety and learners’ sociocultural context. However, in addition to the intense academic competition highlighted in previous research (Bux et al., 2019), this study adds a new dimension, such as maladaptation to the academic transition and the disconnection of curricula setting between secondary and higher education specifically in China’s tertiary institutions. We argue that the transition from secondary schools to tertiary institutions creates new pressures, which can exacerbate students’ FLLA. Similarly, students face challenges when transitioning to new environments with different educational expectations, teaching methods as well as language demands. The maladaptation can lead to heightened stress since students are unprepared for the more advanced language requirements of tertiary studies.

Another finding in this study is the relationship between SRL and FLLA induced by these external sociocultural factors. Students suffering from FFLA are shown to struggle with cognitive overload, time management, and low self-efficacy, all of which are common symptoms of difficulties in self-regulation.

Unlike the studies by Dewaele and MacIntyre (2014) and Li et al. (2020), which proved that higher language proficiency is associated with reduced anxiety, this study found that, regardless of their English proficiency, students at different levels of tertiary institutions suffer from anxiety due to cognitive overload. In order to remain competitive in high-stakes academic environments, where success in English is closely tied to future academic opportunities, students raise their self-expectations, which widens the gap between their cognitive demands and language proficiency, and increases time management challenges. Under the influence of anxiety, students weaken their learning will and self-efficacy. These factors, in turn, further intensify language learning anxiety.

While much of the existing research focused on the symptoms and effects of language learning anxiety (MacIntyre and Gardner, 1991), this study contributes to the field by exploring the deeper mechanisms behind FLLA. Previous studies have identified the external factors contributing to language learning anxiety, such as instruction methods and classroom dynamics (Young, 1991). However, few studies explored the interaction between FLLA and students’ internal cognitive processes such as self-regulation and self-efficacy. This study addresses this gap by showing that the sociocultural factors create a psychological burden which makes learners’ self-regulated learning increasingly difficult.

This study contributes to the existing research in terms of the following three aspects. First, it addresses the problem of homogeneous research population in previous studies, which often restrict research to subjects from a single institution or region. This study examines students from a variety of institutions across different regions of China. This diversity offers a more comprehensive understanding of FLLA’s multifaceted nature. Second, it employs a novel research method. While previous works have utilized quantitative or survey-based approaches, this study introduces grounded theory to investigate FLLA. This approach provides a novel perspective for exploring complex psychological phenomena in language learning. Lastly, it provides a deeper exploration of the content. Prior research has mainly shed light on factors such as anxiety symptoms and intervention measures. This study, however, delves into the underlying mechanisms of FLLA. It offers a new framework for understanding how external pressures shape students’ internal cognitive and emotional experiences, addressing gaps in our understanding the latent aspects of this issue.

## 6 Conclusion

Despite efforts to ensure comprehensive data collection and adherence to theoretical saturation, the reliance on students’ self-reports of anxiety introduces a potential source of bias. Self-reports are inherently subjective. This could affect the reliability of the findings, as students might underreport or overreport their anxiety levels based on personal or cultural factors. On the other hand, the study does not explore how other emotional factors (such as burnout or enjoyment) may interact with anxiety to affect students’ learning experiences. Focusing solely on FLLA without considering these other variables may provide a limited understanding of the broader emotional landscape.

Future research should adopt dynamic complexity theory, such as process tracing and retrospective qualitative modeling, and employ mixed research methods to explore trends in anxiety among Chinese university students and its interactions with other emotional factors. This approach will contribute to a more comprehensive understanding of the trajectory of anxiety and provide a scientific basis for developing more effective intervention strategies.

## Data Availability

The original contributions presented in the study are included in the article/supplementary material, further inquiries can be directed to the corresponding author.
